# Pseudoaneurysma of the internal carotid artery caused by a gunshot wound

**DOI:** 10.1186/1749-8090-8-S1-P82

**Published:** 2013-09-11

**Authors:** Dragan Piljić, Mustafa Tabaković, Amir Tursunović

**Affiliations:** 1 UKC Tuzla - Cardiovascular Surgery Department, Tuzla, Bosnia and Herzegovina

## 

A 22 year old man was admitted in the ICU because of a gunshot wound. At the moment he was admitted he was conscious and breathing spontaneously. On the left side of the lower jaw the entry hole of the gunshot wound was determined. The exit hole was on the right side of the neck. An emergent CT scan of the head and neck was done. On that scan a gunshot wound was established that was spreading from left and forward to the right and back. In the area of the upper jaw's alveolar processus a metal like density area was described. After clinical and laboratory finding were stabilised we decided to perform a third surgery using an interpositing reversed vena saphena magna graft. From the third surgery on, the postoperative cours was proceeding without any further complications. On the second postoperative day the patient was discharged from the ICU and he was moved to the department. He was discharged home. We followed up the patient’s health status in regular time intervals. The findings of a control CT angiography was regular beside the ligation of the external carotid artery.

Three years after he was wounded the patient was examined by a cardiovascular surgeon. There were no significant abnormalities.

